# Supplementary material for: Activation and Functions of Col6a1+ Fibroblasts in Colitis-Associated Cancer
Source: Int J Mol Sci. 2023 Dec 21;25(1):148. doi: 10.3390/ijms25010148 (PMC10778587; doi:10.3390/ijms25010148)
Supplement: Supplementary file 1 [file ijms-25-00148-s001.zip › Supplementary Figures.pdf]

Supplementary Figures

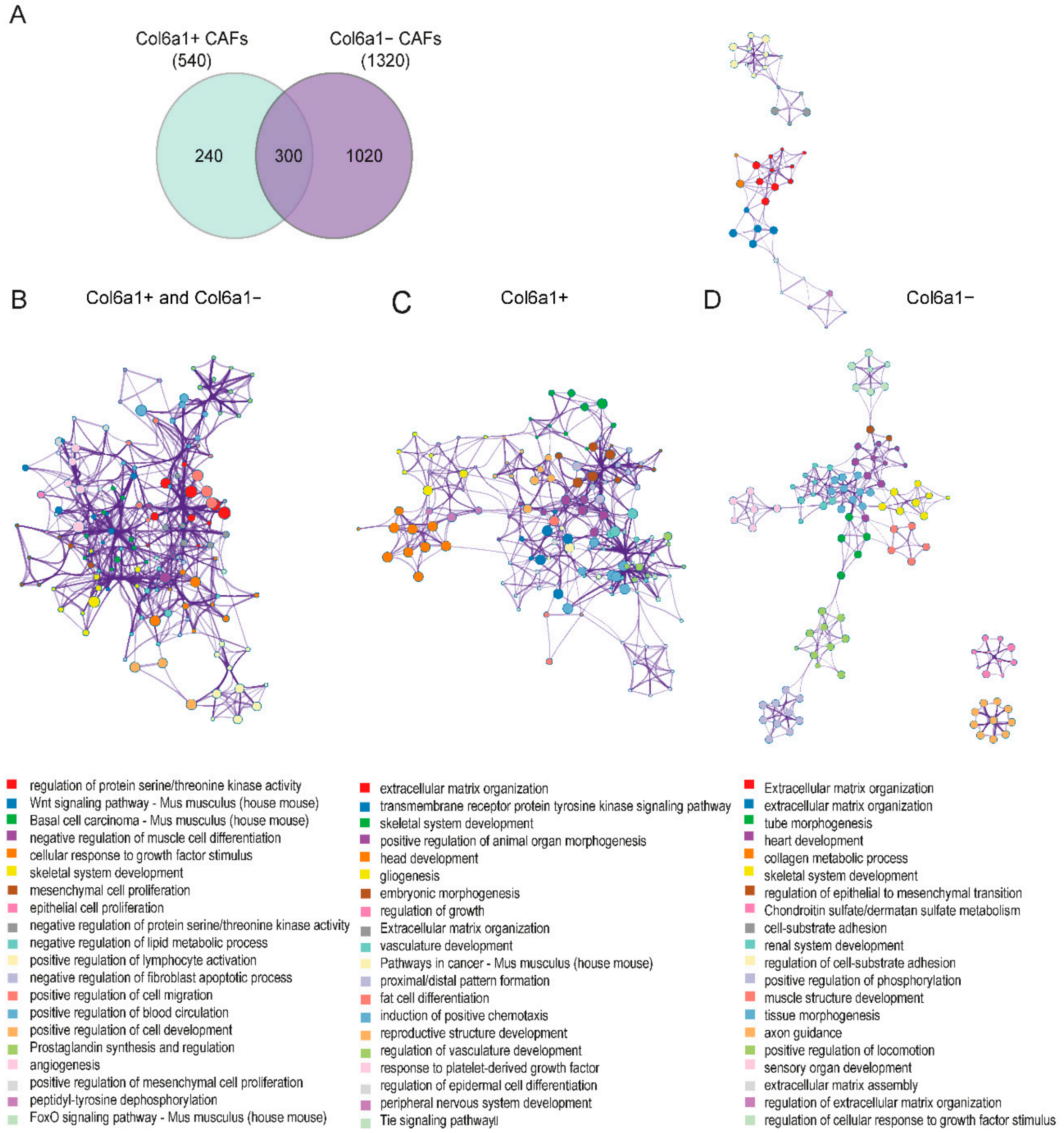

Supplementary Figure S1. Col6a1+ and Col6a1− CAFs are activated upon AOM/DSS colon carcinogenesis. (**A**) Venn diagram showing the differential and common downregulated genes in Col6a1+ and Col6a1− CAFs. (**B**) Network of enriched terms in Col6a1+ CAF and Col6a1− CAF common downregulated gene signature. (**C**) Network of enriched terms in Col6a1^{+} CAF unique downregulated gene signature. (**D**) Network of enriched terms in Col6a1− CAF unique downregulated gene signature. Networks are colored by cluster ID, where nodes that share the same cluster ID are typically close to each other (generated through metascape.org).

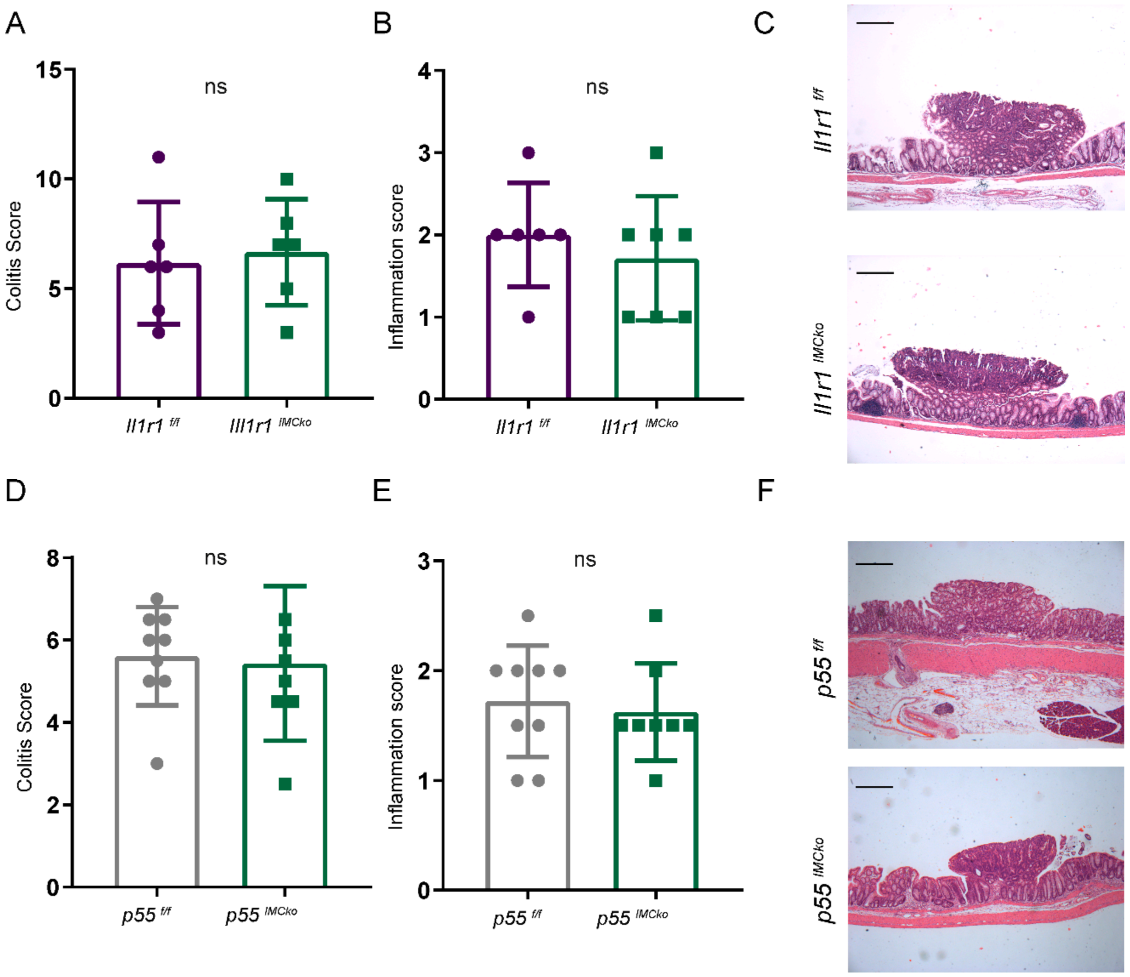

Supplementary Figure S2. Deletion of IL−1R1 and TNF−R1 in Col6a1+ IMCs does not affect colitis or inflammation score. (**A**) Colitis score and (**B**) Inflammation score per mouse, and (**C**) representative H/E pictures of adenomas *in Il1r1*^{IMCko} mice (n = 6) and their littermate controls (n = 6) at the end of the AOM/DSS protocol (one representative experiment of four performed). (**D**) Colitis score and (**E**) Inflammation score per mouse and (**F**) representative H/E pictures of adenomas in *p55*^{IMCko} mice (n = 8) and their littermate controls (n = 9) at the end of the AOM/DSS protocol (one representative experiment of two performed). ns, not statistically significant. Scale bar = 1mm
